# Gibberellin-Producing Bacteria Isolated from Coastal Soil Enhance Seed Germination of Mallow and Broccoli Plants under Saline Conditions

**DOI:** 10.3390/biotech12040066

**Published:** 2023-12-11

**Authors:** Ji-In Woo, Md. Injamum-Ul-Hoque, Nazree Zainurin, Shifa Shaffique, Eun-Hae Kwon, Ho-Jun Gam, Jin Ryeol Jeon, In-Jung Lee, Gil-Jae Joo, Sang-Mo Kang

**Affiliations:** 1 Department of Applied Biosciences, Kyungpook National University, Daegu 41566, Republic of Korea; wjxsj99@naver.com (J.-I.W.); mdinjamum92@knu.ac.kr (M.I.-U.-H.); nazreezainurin@hotmail.com (N.Z.); shifa.2021@knu.ac.kr (S.S.); eunhaekwon@naver.com (E.-H.K.); jeff4237@naver.com (H.-J.G.); 98micael10@naver.com (J.R.J.); ijlee@knu.ac.kr (I.-J.L.); 2 Institute of Agricultural Science and Technology, Kyungpook National University, Daegu 41566, Republic of Korea; gjjoo@knu.ac.kr

**Keywords:** salt stress, plant growth–promoting rhizobacteria, antioxidants, percent germination, germination performance index

## Abstract

Salinity hinders plant growth, posing a substantial challenge to sustainable agricultural yield maintenance. The application of plant growth-promoting rhizobacteria (PGPR) offers an emerging strategy to mitigate the detrimental effects of high salinity levels. This study aimed to isolate and identify gibberellin-producing bacteria and their impact on the seed germination of *Malva verticillata* (mallow) and *Brassica oleracea var. italica* (broccoli) under salt stress. In this study, seven bacterial isolates (KW01, KW02, KW03, KW04, KW05, KW06, and KW07) were used to assess their capacity for producing various growth-promoting traits and their tolerance to varying amounts of salinity (100 mM and 150 Mm NaCl). The findings revealed that KW05 and KW07 isolates outperformed other isolates in synthesizing indole-3-acetic acid, siderophores, and exopolysaccharides and in solubilizing phosphates. These isolates also enhanced phosphatase activity and antioxidant levels, including superoxide dismutase and catalase. Both KW05 and KW07 isolate highlight the growth-promoting effects of gibberellin by enhancing of growth parameters of Waito-C rice. Further, gas chromatography–mass spectrometry validation confirmed the ability of KW05 and KW07 to produce gibberellins (GAs), including GA_1_, GA_3_, GA_4_, and GA_7_. Seed germination metrics were enhanced due to the inoculation of KW05 and KW07. Moreover, inoculation with KW05 increased the fresh weight (FW) (7.82%) and total length (38.61%) of mallow under salt stress. Inoculation with KW07 increased the FW (32.04%) and shoot length of mallow under salt stress. A single inoculation of these two isolates increased broccoli plants’ FW and shoot length under salt stress. Gibberellin-producing bacteria helps in plant growth promotion by improving salt tolerance by stimulating root elongation and facilitating enhanced absorption of water and nutrient uptake in salty environments. Based on these findings, they can play a role in boosting agricultural yield in salt-affected areas, which would help to ensure the long-term viability of agriculture in coastal regions.

## 1. Introduction

In the present century, humanity faces two critical challenges: shifting climatic patterns and global food security concerns [1]. Climate change is causing a steady rise in salinity on farmland, which could have disastrous global consequence [2]. Globally, soil salinization could pose the risk of losing over half of agricultural land [3,4]. The Food and Agriculture Organization (FAO) has reported that nearly 75% of the world’s land area, comprising 424 million hectares of topsoil (0–30 cm) and 833 million hectares of subsurface (30–100 cm), are affected by high salinity levels [5]. During this period of excessive soil salts, plant growth and development are stunted leading to a state of saline stress of plants [6]. This state directly causes a downturn on crop physiological and metabolic processes, evident in stunted seed germination, poor seedling development, reduced photosynthesis, increased ion toxicity, augmented water stress, and decreased protein and lipid metabolism rates [7,8,9]. Also, cellular osmotic equilibrium is disturbed [10] and reactive oxygen species (ROS) are synthesized, escalating plants oxidative stress. Ion toxicity and water deficiencies caused by soil salinity, often due to NaCl, may hinder plant development [11]. Plants respond with physiological reactions to unfavourable environmental conditions, enhancing resilience through signal transduction, stress-responsive gene activation or suppression, functional protein modulation, and changes in specific cellular organelles, particularly chloroplasts, mitochondria, and peroxisomes [12,13,14,15]. Plants also store more osmolytes, such as proline and glycine betaine, for defence against salt stress [15,16]. Under such stress, plants synthesize nonenzymatic compounds, including phenolics, flavonoids, and glutathione [15]. Furthermore, they induce enzymatic antioxidants production, such as peroxidase and catalase, alongside enzymes integral to the ascorbate–glutathione cycle [17,18,19].

Conventional agricultural practices, exemplified by chemical-based fertilizer and pesticide use [20], have major drawbacks, e.g., harming the environment, carrying substantial costs, and impacting human well-being. Alternatively, breeding and genetic modification methods are time consuming, costly, and restricted by societal approval. In contrast, microbial–plant associations hold potential to enhance plant resilience against the detrimental impact of salinity stress [21]. Particularly, plant growth–promoting rhizobacteria (PGPR) have garnered attention, as they elevate both crop growth and nutrient levels, ultimately resulting in the enhancement of crop yields [22]. Specifically, bacteria which can grow in salt conditions are equipped with growth-promoting attributes. These attributes are pivotal in enabling plant growth within saline environments [23]. Beyond economic gains, these PGPRs also reduce salt absorption, showcasing their multifaceted benefits [24]. Bacterial endophytes adeptly inhabit plant tissues without inducing visible pathogenic symptoms. PGPRs have the ability to orchestrate phytohormone and enzyme production, as well as complex nutrient mobilization and translocation processes. They perform important functions such as generating ammonia, fixing nitrogen, and solubilizing phosphate. Additionally, PGPRs have a remarkable capacity for producing various secondary metabolites, including siderophores, cyclic lipopeptides, effectors, antibiotics, and plant hormones [25,26,27,28]. These dynamic processes constitute the foundational basis for essential adaptations in response to severe salinity stress conditions [29,30,31,32].

Gibberellins are a class of tetracyclic diterpenoid carboxylic acids, named after *Gibberella fujikuroi*, a fungus which is currently known as *Fusarium fujikuroi*. Initially, these compounds were isolated due to their ability to elongate plant shoot [33]. Gibberellins govern shifts between plant growth and development stages, including processes such as cell division and elongation [34]. Key bioactive gibberellins include the gibberellic acids (GAs) GA_1_, GA_3_, GA_4_, and GA_7_, with GA_1_ and GA_4_ prevalent across various plants [35]. The gibberellin signalling pathway is usually suppressed during stress conditions, such as high salinity level and deprivation of water and flood. This enables growth adjustment according to the environment, preventing excessive ROS accumulation [34,36].

*Malva verticillata* (mallow) possesses diverse chemical constituents, such as polysaccharides, coumarins, polyphenols, flavonoids, vitamins, terpenes, and tannins. These compounds are primarily concentrated in various plant organs, with leaves and flowers being prominent sources. These constituents intricately link to the biological effects of mallow plants. In a broader context, *Malva* spp. demonstrates moderate antimicrobial potential, significant anti-inflammatory and wound healing properties, potent antioxidant capabilities, and promising anticancer attributes. However, clinical trials are needed to explore the efficacy of *Malva* spp. in treating various human health issues, as indicated by in vitro and in vivo test results [37]. *Brassica oleracea* (broccoli), part of the cruciferous vegetable family, has widespread culinary utilization and maintains a prominent role in traditional medicinal practices across diverse cultures. Its notable bioactive constituents include glucosinolates, polyphenols, and vitamins, known for their health-promoting potential. Particularly well-documented are its anticancer, antioxidant, anti-inflammatory, and cardioprotective properties [38]. Keeping this view, the aim of this study was designed to isolate and identify gibberellin-producing bacteria and their impact on the seed germination of *Malva verticillata* (mallow) and *Brassica oleracea var. italica* (broccoli) under salt stress. These findings may play a role in boosting agricultural yield in salt-affected areas, which would help to ensure the long-term viability of agriculture in coastal regions. 

## 2. Materials and Methods

### 2.1. Collection and Isolation of Bacteria from Rhizospheric Soil

To isolate saline-resilient microbes, a total of 50 soil samples of approximately 20 g of each were randomly collected from plant rhizospheres (10cm depth) at Pohang Beach, Republic of Korea in 2019. The samples were then transported to Kyungpook National University’s Crop Physiology Laboratory in Daegu, Republic of Korea, while maintaining the temperature of the samples at 0–6 °C with ice. In the laboratory, each 1 g soil sample was immersed in a 0.85% NaCl solution. Dilutions ranging from 10^−4^ to 10^−6^ were prepared, and 100 μL of this suspension was evenly spread on a Petri dish. Subsequently, incubation at 30 °C was conducted for 7 days in a controlled environment. After cultivation, individual colonies were meticulously selected and replated on trypticase soy agar media. This isolation process yielded a total of 290 microorganisms from the soil samples, all of which exhibited various confirmed growth-promoting attributes. Following thorough evaluation, a final subset of seven bacterial strains was chosen for further investigation based on four effectiveness criteria: (i) Salkowski test, (ii) siderophore production, (iii) phosphate solubilization, and (iv) exopolysaccharides (EPS) production, which were met by these selected strains.

### 2.2. Screening Bioassay for Growth Promotion in Waito-C Rice Seeds

The inoculation of isolates to GA-deficient, mutant rice cultivar Waito-C was conducted to observe the isolates potential to stimulate the growth of the mentioned cultivar. First, the rice seeds were sterilized and soaked in the broths of the selected seven bacterial isolates (10^8^ cfu/mL), whereas control seedlings were soaked with autoclaved deionized water. After soaking for 6 h in a shaking incubator, the bacteria-inoculated seeds were placed in a Petri dish containing 0.8% agar-media. The seeds were germinated and grew for 7 days in a regulated condition (14 h/10 h light/dark, 28 °C/24 °C, 70% relative humidity, 250 µmol m^−2^ s^−1^ light intensity). Growth metrics of the plants were observed and recorded 7 days after sowing. 

### 2.3. Experiment Location, Method, and Design

The research was carried out under a controlled greenhouse situated at Kyungpook National University in Daegu. Isolates KW05 and KW07 were let grown in Tryptic Soy Broth for 5 days at 30 °C in a shaking incubator operating at 200 rpm. First, bacterial suspension (cells) was added with autoclaved deionized water to get a diluted suspension of 10^8^ CFU/mL. Mallow (var. Chungchima) and broccoli (var. Sorrento) seeds were collected from Danong (Gyeonggi, Republic of Korea). The seeds were surface removed of their impurities and sterilized with 2.5% NaOCl for 10 min before rinsing with autoclaved double-deionized water. Then, these seeds were sown into plastic trays filled with horticultural soil and allowed to grow in a greenhouse with controlled environment at 30 ± 2 °C. The horticulture soil was autoclaved three times and consisted of varying proportions of major components: peat moss (13–18%), perlite (7–11%), coco-peat (63–68%), and zeolite (6–8%). The soil macronutrient content included approximately 90 mg/kg NH_4_^+^, 205 mg/kg NO_3_^−^, 350 mg/kg P_2_O_5_, and 100 mg/kg K_2_O. The pH level of the soil ranged from 5 to 7, with an electrical conductivity (S/m) of ≤1.2. After one week of growth, seedlings were transplanted to pots (10 cm × 9 cm) and inoculated with 5 mL of bacterial culture via the soil drenching method. Following a 5-day wait, 100 mM NaCl was added. Germination metrics and plant growth characteristics were recorded after salt stress was initiated.

### 2.4. Screening for Indole-3-Acetic Acid Production

Pure bacterial isolates were combined with Salkowski’s reagent (50 mL of 35% HClO_4_, 1 mL of 0.5 M FeCl_3_) at a 1:1 ratio. The reaction required a no-light environment for 0.5 h, and a hue shift from white to pink indicated the presence of indole-3-acetic acid (IAA) [39].

### 2.5. Screening for Siderophore Production

To evaluate siderophore production, an agar diffusion assay was performed. In this process, 2 μL of pure bacterial culture was carefully introduced onto Petri dish containing agar-based media supplemented with Chromeazurol S. Then, isolates grown at 25–30 °C for 3 days, to allow for the formation of orange halos surrounding the bacterial colonies, which indicates siderophores production [40]. The measurements of the halos were assessed.

### 2.6. Screening for Phosphate Solubilization Activity

Phosphate-solubilizing capability exhibited by bacterial isolates was investigated, following established procedural guidelines [41] with slight modifications. Phosphate-solubilizing media was prepared, comprising glucose (10 g/L), (NH_4_)_2_ SO_4_ (0.5g /L), NaCl (0.3 g/L), KCl(0.3 g/L), FeSO_4_ (0.03 g/L), MgSO_4_ (0.3 g/L), MnSO_4_ (0.03 g/L), Ca_3_(PO_4_) (5 g/L), and agar (20 g/L) before autoclaving for 1 h. A small amount (1 µL) of pure bacterial culture was cultivated on Trypticase Soy Agar medium enriched with tricalcium phosphate [Ca_3_(PO4)_2_] and was transferred to the above-mentioned media. The resulting culture was then incubated at 25−30 °C for 7 days. In order to assess the isolates’ phosphate solubilization capability, the plates were consistently examined, focusing on the appearance of transparent halos encircling bacterial populations. These halos served as discernible indicators of the isolates’ potential to solubilize phosphorus.

### 2.7. Screening for Exopolysaccharides (EPS)

To identify potential EPS production by bacterial isolates, the Congo red agar plate test was utilized. The media of the assay comprised of Luria–Bertani (LB) broth (18 g/L), agar (2%), sucrose (5%), and Congo red (0.8 g/L). This mixture was thoroughly blended and subsequently autoclaved for sterilization, before moulding onto plates. After plate preparation, microbial entities were cultivated onto it for 5 days at 30–35 °C. Confirmation of EPS presence relied on visually assessing colonies appearing black against a red background due to their interaction with iron, which substantiated their production [42,43].

### 2.8. Quantification of Indole-3-Acetic Acid Production (IAA)

To quantify the in vitro IAA production by these seven bacterial isolates through GC/MS-SIM analysis, the bacterial isolates were cultured in LB media for a duration of 3 days. The LB medium was composed of 10 g of tryptone, 5 g of yeast extract, and 10 g of NaCl, with a pH level of 7.0; it was autoclaved for 15 min at 121 °C. Then, the medium was subjected to centrifugation at 5000× *g* for a period of 15 min to separate the cells from the culture broth. In accordance with the methodology described by Lubna et al. [44], each individual culture broth was subsequently subjected to an analysis of IAA production. To determine the IAA concentration in these broths, the IAA peaks were compared against those of a known standard and this was achieved using using gas chromatography/mass spectrometry (GC/MS) in the selected ion-monitoring mode (SIM).

### 2.9. Identification and Phylogenetic Analysis

The taxonomic classification of isolated bacterial strains relied on analysing a partial segment of their 16S ribosomal RNA gene (rRNA). To achieve this, the 16S rRNA gene was polymerase chain reaction amplified, sequenced, and then identified using the BLAST algorithm, comparing against sequences in the NCBI database. The 16S rRNA was amplified using universal primer pairs: 5′-AGAGTTTGATCACTGGCTCAG-3′ and 1492R (5′-CGGCTTACCTTGTTACGACTT-3′) [45,46]. The amplification process commenced with an initial denaturation step at a temperature of 95 °C for a duration of 5 min. This was followed by a series of 30 cycles, each consisting of denaturation at 94 °C for 1 min, annealing at 57 °C for 40 s, and primer extension at 72 °C for 1.5 min. Subsequently, the last extension step was conducted at a temperature of 72 °C for a duration of 10 min. The PCR products underwent purification by 1% agarose gel electrophoresis in order to facilitate further sequencing and analysis (SolGent Co., Ltd., Daejeon, Republic of Korea). The highly analogous sequences were aligned using the CLUSTAL-W program in MEGA X (version 7.222) [47]. To construct the phylogenetic tree, the maximum likelihood technique was employed in the MEGA X platform. To enhance the tree’s statistical reliability, 1,000 bootstrap replications were performed, providing robust statistical support for each node in the phylogenetic arrangement. The sequence data obtained were officially deposited in the gene bank under the accessions *Priestia aryabhattai* KW05 (OQ991250) and *Pseudomonas frederiksbergensis* KW07 (OQ991249).

### 2.10. Determination of Acid Phosphatase Activity 

Phosphatase activity determination was conducted using a modified version of a previously established approach [48]. For this assessment, bacterial isolates were inoculated into tryptic soy broth media supplemented with calcium phosphate. After bacterial cultivation, 1 mL of the supernatant was mixed with a solution containing 1 mL of 25 mM p-nitrophenyl phosphate (pNP) and 4 mL of a customized universal buffer. This reaction occurred at 37 °C. After 60 min of incubation, absorbance levels were measured at a wavelength of 410 nm.

### 2.11. Measurement of the Activity of Superoxide Dismutase

Plant samples were ground using a buffer solution [50 mM Tris HCl, 10 mM ethylenediaminetetraacetic acid (EDTA; pH 8.5)], and 1.3 mL of this mixture was utilized. Subsequently, 100 μL of pyrogallol (7.2 mM) was combined with 100 μL of the extracted sample, and this mixture was incubated at 25 °C for 10 min. The reaction was halted by adding 50 μL of 1 N hydrochloric acid and the spectrophotometric absorbance was measured at 420 nm [49]. Superoxide dismutase (SOD) activity was assessed using the following equation:

(1) $$SODactivity\left(\%\right)=\left[1-\left(A-B\right)/C\times 100\right]$$

where A is the pyrogallol-containing extracted sample, B is the pyrogallol-free extracted sample, and C is the pyrogallol-containing buffer solution control.

### 2.12. Measurement of the Activity of Catalase

Catalase activity in plants inoculated with KW05 and KW07 followed the approach outlined by Bilal et al. [50]. Briefly, 200 mg of the leaves were pulverized in a buffer composed of 50 mM Tris HCl (pH 7.0), 3 mM magnesium chloride, 1 mM EDTA, and 1% polyvinylpyrrolidone, in order to obtain the supernatant. Subsequently, a mixture of 0.2 M H_2_O_2_ in a 10 mM phosphate solution (pH 7.0) was added to 0.5 mL of the supernatant. The resulting absorbance was read at 240 nm using a spectrophotometer. Catalase determination was calibrated using standard curves.

### 2.13. Quantification of Gibberellins in the Culture Broth

To quantify gibberellins, the chosen isolates of KW05 and KW07 were cultured in 120 mL of nutrient broth for 7 days at 30 °C with continuous shaking at 120 rpm. After incubation, the cultures underwent centrifugation at 10,000× *g*, followed by filtration through 0.45 µm filter membrane. The resulting culture filtrate (CF) was subjected to GC-MSSIM analysis for gibberellin quantification. This assessment method was adapted from a modified protocol described by Lee et al. [51], using several internal gibberellin standards (17-2H2) obtained from Prof. Lewis N. Mander (Australian National University, Canberra, Australia). Extracts from distinct fractions were analysed on a C18 column (90–130 mm; Waters Corp., Milford, MA, USA). An injection volume of 1 mL was consistently used for each gibberellin type in GC-MS analysis. Quantities of bioactive GAs (GA_1_, GA_3_, GA_4_, and GA_7_) and inactive GAs (GA_8_, GA_9_, GA_12_, GA_19_, GA_20_, GA_24_, and GA_36_) in the CF were calculated based on peak area ratios. A hydrocarbon standard was also integrated to determine retention times in the analysis process.

### 2.14. Screening Bioassay for Growth Promotion in Mallow and Broccoli Seeds

Two bacterial isolates (KW05 And KW07) were inoculated on mallow (var. Chungchima) and broccoli (var. Sorrento) seeds to determine the germination of the above-mentioned seeds. The seeds went through sterilization by soaking in 2.5% NaOCl. After 5 min of soaking, 10 seeds were placed on a double layered, sterile filter paper in a Petri dish. An inoculation of each bacterial isolate (5 mL) along with NaCl (100 mM) were applied to the seeds before keeping them in an incubator with defined settings for 5 days (h/10 h light/dark, 28 °C /24 °C, 70% relative humidity, 250 µmol m^−2^ s^−1^ light intensity). The germination metrics of mallow and broccoli seedlings were measured five days after germination.

### 2.15. Measurement of Germination Metrics

The percent germination, mean germination time, germination rate, germination performance index, germination energy, and mean daily germination of mallow and broccoli plants were recorded after 5 days of treatment. The above-mentioned germination parameters were calculated using the following formulas [52]:

(2) $$PG\left(\%\right)=\frac{N}{S}\times 100$$

where PG is percent germination, N is the total number of germinations, and S is the total number of seeds [52];

(3) $$GE\left(\%\right)=\frac{n}{S}\times 100$$

where GE is germination energy, n is the number of germinated seeds on 3rd day, and S is the total number of seeds [52];

(4) $$GR=\sum \frac{Ni}{Ti}$$

where GR is the germination rate, Ni is the number of germinations at the day of assessment, and Ti is the number of days after sowing [53],

(5) $$MDG=\frac{N}{T}$$

where MDG is the mean daily germination, N is the total number of germinations, and T is the total number of days in the investigation [54];

(6) $$MGT=\sum \frac{\left(TixNi\right)}{N}$$

where MGT is the mean germination time, Ti is the number of days after sowing, Ni is the number of germinations at the day of assessment, and N is the total number of germinations [55];

(7) $$GPI=\frac{Percent\;germination\;(PG)}{Mean\;germination\;time\;(MGT)}$$

where GPI is the germination performance index.

### 2.16. Statistical Analysis

A completely randomized design was employed for all experiments involving the administration of bacterial cultures to plants and the assessment of PGPR effects. Each test consisted of three replicates. Analysis of variance (ANOVA) followed by Duncan’s multiple range test at a significance level of *p* ≤ 0.05 revealed significant variations in mean values, with SAS 9.1 statistical software used to conduct these tests. GraphPad Prism (version 5.0; San Diego, CA, USA) was used to generate graphical representations.

## 3. Results

### 3.1. Initial Screening for PGPR Characteristics in Isolates

To isolate bacteria, soil samples were collected from diverse longitudes and latitudes (Table 1). The ability to produce IAA, siderophores, and EPS as well as to solubilize phosphate guided the selection of seven bacterial isolates, namely KW01, KW02, KW03, KW04, KW05, KW06, and KW07. Among these isolates, KW05 and KW07 emerged as highly effective during the primary screening (Table 1).

### 3.2. Effects of Bacterial Isolates on IAA Content, Phosphatase Activity, and Antioxidant Levels

**Figure 1:**
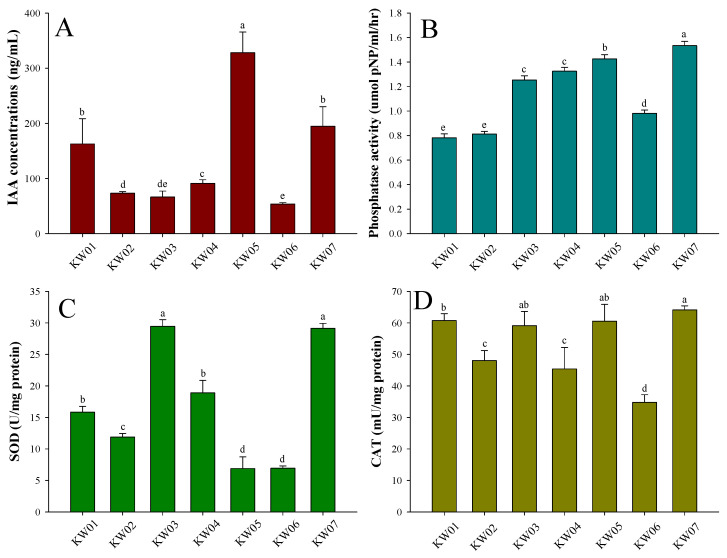
Comparative analysis of (**A**) IAA production, (**B**) phosphatase activity, (**C**) superoxide dismutase (SOD) activity, and (**D**) catalase activity among seven bacterial isolates cultured in LB medium. Data represent means ± standard deviations (SDs) from three replicates. Shared letters denote statistical similarity, whereas different letters indicate significant differences followed by Duncan´s multiple range test at a significance level of *p* ≤ 0.05.

The bacterial isolates KW01, KW02, KW03, KW04, KW05, KW06, and KW07 exhibited a diverse range of IAA-production rates (Figure 1A), with IAA being a critical regulator of growth and development. Notably, KW05 emerged as a prominent producer, yielding a substantial IAA content of 328.21 ng/mL. Following this, KW07 demonstrated IAA production of 194.79 ng/mL. In contrast, KW06 exhibited the lowest IAA synthesis (53.75 ng/mL). In addition, KW01, KW02, KW03, and KW04 exhibited IAA production levels of 162.69, 73.40, 66.47, and 91.14 ng/mL, respectively.

The seven bacterial isolates displayed varying degrees of phosphatase activity (Figure 1B). Notably, KW07 exhibited the highest phosphatase activity, with a rate of 1.53 µmol/pNP/mL/h, followed by KW05, with a rate of 1.425 µmol/pNP/mL/h. KW01, KW02, KW03, KW04, and KW06 exhibited phosphatase activities of 0.72, 0.81, 1.25, 1.33, and 0.98 µmol/pNP/mL/h, respectively. Figure 1C shows the distinct SOD activity among the seven bacterial isolates. Notably, KW03 and KW07 showed robust SOD activity and statistically similar production rates, yielding 29.45 and 29.15 U/mg protein, respectively. In contrast, KW01, KW02, KW04, KW05, and KW06 exhibited SOD activities of 15.85, 11.90, 18.90, 6.90, and 6.95 U/mg protein, respectively. The activity of catalase across distinct bacterial isolates is presented in Figure 1D. Notably, KW07 yielded a higher catalase activity (64.12 U/mg protein) in contrast to KW06, which exhibited notably low catalase activity (34.8 U/mg protein). KW01, KW02, KW03, KW04, and KW05 showed catalase activities of 60.75, 48.05, 59.15, 45.4, and 60.55 U/mg protein, respectively.

### 3.3. Screening for Salt Tolerance

**Figure 2:**
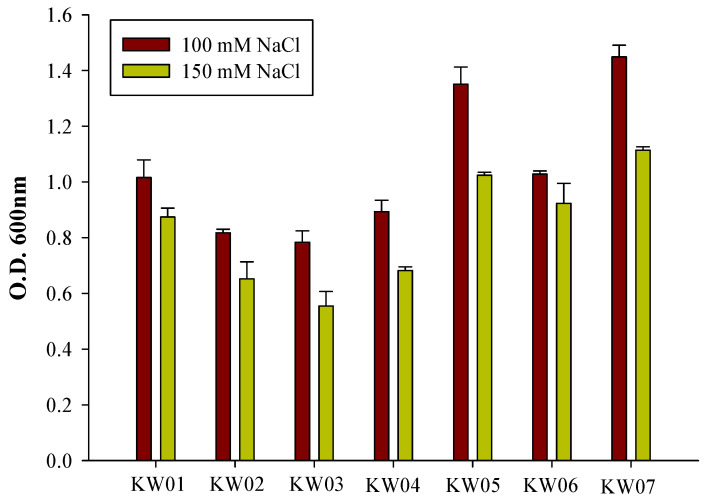
Assessment of the PGPR activity of bacterial isolates using optical density (OD) at 600 nm under two NaCl concentrations (100 and 150 mM). Data represent means ± SDs of three replicates.

The seven bacterial isolates underwent in vitro analysis to assess their tolerance to salt stress across varying NaCl concentrations (Figure 2). The growth behaviour of the isolates, as quantified via optical density (OD) measurements at 600 nm, is shown in Figure 2. Notably, KW07 showed the most pronounced tolerance to 100 mM NaCl, with KW05 also exhibiting a high degree of resilience. However, as the NaCl concentration was increased to 150 mM, these two bacterial isolates exhibited a substantial decline in growth.

### 3.4. Effects of Bacterial Inoculation on Waito-C Rice Plants

**Figure 3:**
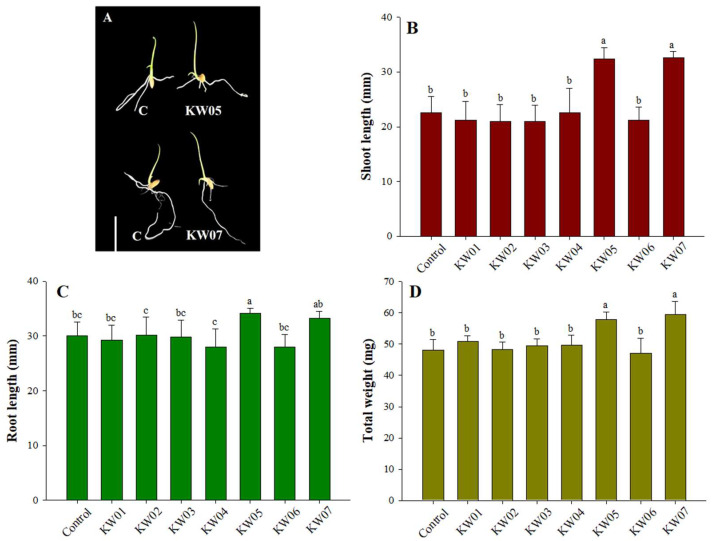
Effects of seven bacterial isolates on the growth attributes of gibberellin-deficient Waito-C rice plants. Data represent means ± SDs of three replicates. Shared letters denote statistical similarity, whereas different letters indicate significant differences followed by Duncan’s multiple range test at a significance level of *p* ≤ 0.05. In figure (**A**), the bar represents 1 cm.

Inoculation of the seven bacterial isolates enhanced both root and shoot lengths of Waito-C rice plants, as well as total plant fresh weight (Figure 3A). Among the isolates, KW05 and KW07 were notable contributors, fostering marked increases in shoot length of 43.36% and 44.24%, respectively, compared with the control condition (Figure 3B). Similarly, KW05 and KW07 facilitated 14% and 10% augmentations in root length, respectively, in comparison to the control (Figure 3C). Cumulative plant weight was also favourably influenced by KW05 and KW07, with 20.00% and 23.75% growth rates observed following inoculation, respectively (Figure 3D). 

**Figure 4:**
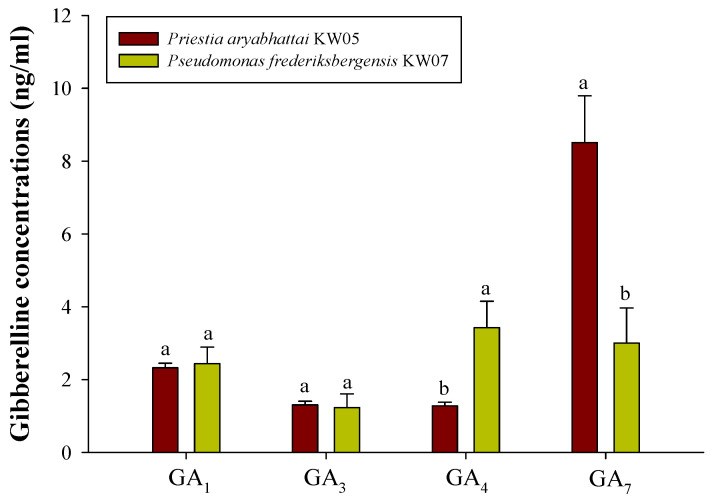
Gibberellic acid biosynthesis potential of two bacterial isolates. Data represent means ± SDs of three replicates. Shared letters denote statistical similarity, whereas different letters indicate significant differences followed by Duncan´s multiple range test at a significance level of *p* ≤ 0.05.

Collectively, these results underscore the beneficial impact of PGPR isolates, with KW05 and KW07 most effectively enhancing the accumulation of GAs compared with other isolates. These results were confirmed through GC/MS analysis of KW05 and KW07’s capacity to generate GAs. Figure 4 shows the synthesis of endogenous GA_1_, GA_3_, GA_4_, and GA_7_ after the inoculation of KW05 and KW07. Notably, KW05 showed higher production levels, particularly of GA_7_, which reach 8.5078 ng/mL. GA_7_ was followed by GA_1_ (2.32 ng/mL), GA_3_ (1.30 ng/mL), and GA_4_ (1.27 ng/mL). KW07 exhibited a different trend, with GA_4_ produced most abundantly, reaching 3.42 ng/mL, followed by GA_7_ (3.00 ng/mL), GA_1_ (2.44 ng/mL), and GA_3_ (1.23 ng/mL). These findings underscore the differences in the gibberellin production profiles of KW05 and KW07.

### 3.5. Molecular Identification of Bacterial Isolates

**Figure 5:**
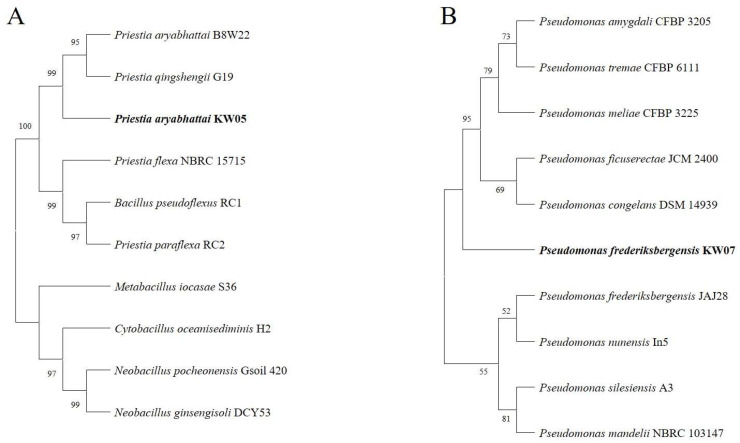
Phylogenetic dendrograms, constructed using partial 16S rRNA nucleotide sequences, depicting the relationship between the bacterial isolates KW05 and KW07 and their closely related taxa. (**A**) *Priestia aryabhattai* (KW05); (**B**) *Pseudomonas frederiksbergensis* (KW07). Numbers at nodes represent bootstrap values for 1000 replicates determined by neighbour-joining method.

Bacterial isolates’ partial 16S rRNA gene sequences were analysed using the BLAST program, leading to the retrieval of the closest strain sequences for phylogenetic tree construction (Figure 5). Specifically, KW05 exhibited a 99% identity match with *Priestia aryabhattai* (Figure 5A), whereas KW07 showed a 99% similarity to *Pseudomonas frederiksbergensis* (Figure 5B). These sequence matches were formally submitted to NCBI under the accession numbers KW05 OQ991250 and KW07 OQ991249, respectively. For phylogenetic tree construction, the neighbour-joining method was used to effectively showcase the evolutionary relationship among these isolates.

### 3.6. Influence of KW05 and KW07 on the Growth Characteristics of Mallow and Broccoli

**Figure 6:**
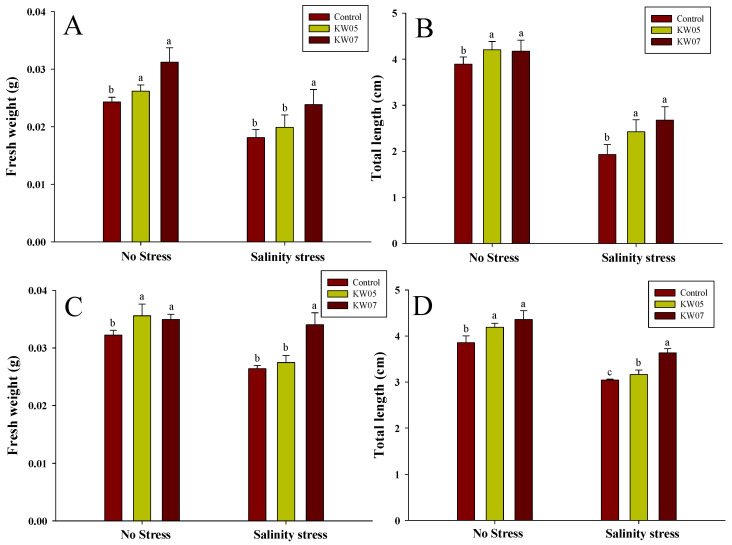
Effects of KW05 and KW07 inoculation on the growth characteristics of mallow (**A**,**B**) and broccoli (**C**,**D**) seedlings under no- and salt-stress conditions. Data represent means ± SDs from three replicates. Shared letters show statistical similarity, whereas different letters indicate significant differences followed by Duncan’s multiple range test at a significance level of *p* ≤ 0.05.

Growth augmentation resulting from PGPR inoculation in *M. verticillata* (mallow) and *B. oleracea* var. *italica* (broccoli) was seen in both normal and stress-induced conditions (Figure 6). Bacterial inoculation with *M. verticillata* led to significant improvements in fresh weight and total length under non-stressed conditions compared with uninoculated plants (*p* < 0.05) (Figure 6A,B). Under saline stress, KW07 and KW05 inoculation yielded 32.04% and 7.82% increases in fresh weight, respectively, compared with uninoculated plants. Under the same stress conditions, KW05 and KW07 inoculation evoked significant increases in the total length of *M. verticillata* plants, with 38.61% and 25.50% enhancements observed, respectively (Figure 6B).

A similar trend was observed in broccoli plant under saline-induced stress. Inoculation with KW07 resulted in a substantial 28.79% fresh weight increase, whereas KW05-inoculated plants exhibited a notable 4.17% increase, both compared with uninoculated plants. In addition, KW07 inoculation yielded a 19.34% increase in total length, whereas KW05 inoculation led to a 3.9% increase (Figure 6C,D). Collectively, these findings underscore the potential of KW05 and KW07 inoculation to significantly elevate growth and bolster stress tolerance in both mallow and broccoli plants.

Table 2 represents the germination metrics (PG, GE, GR, MGT, MDG, and GPI) of mallow and broccoli seedlings under non-saline and saline conditions, with and without inoculation of KW05 and KW07. Under saline conditions, the aforementioned characteristics were adversely affected. However, the inoculation of KW05 on mallow plants mitigated these adverse effects, resulting in significant increases in GE, GR, MDG, and GPI of 40%, 21%, 19%, and 17%, respectively, as well as significant shortening of MGT by 7%, compared with uninoculated plants (*p* < 0.05). Regarding broccoli, the inoculation of KW07 protected against the detrimental effects of saline stress. It led to significant increases in GR, MDG, and GPI by 27%, 4%, and 50%, respectively, as well as significant shortening of MGT by 32%, compared with uninoculated broccoli. In summary, despite mallow and broccoli plants facing adverse conditions due to high salinity levels, KW05 and KW07 inoculation alleviated these negative effects and promoted more favourable germination conditions.

## 4. Discussion

Saline conditions severely hinder plant growth and can even lead to plant death. The initial phase of germination, water imbibition, is susceptible to various factors, such as reduced seed water uptake, under these conditions [56]. These challenges encompass excessive sodium and chloride ion uptake, which can cause cellular and molecular damage to seeds [57,58]. In this study, KW05 and KW07 produced significant amount of IAA, gibberellin, antioxidant, as well as germination indices of mallow and broccoli plants under salt stress compared to the control. Table 1 highlights isolates KW05 and KW07 as producers of metabolites crucial for surviving high saline conditions. Siderophores, iron-chelating agents, regulate iron availability to plants. For example, they enhance germination and seedling vigour in cucumber, tomato, chickpea, pigeon pea, and groundnuts [59]. Imbibition initiation during germination is facilitated by potassium [60]. Inoculating potassium-solubilizing bacteria promotes germination in groundnuts under saline conditions [61]. EPS ensures plant survival during stress, particularly under saline conditions, even protecting the PGPR when conditions are unfavourable for growth. The EPS matrix creates a biofilm that captures Na^+^ ions and prevents ion influx as well as retaining water [62,63]. In the present study, the antioxidant levels of the isolates were measured (Figure 1). Excessive ROS during saline conditions are well-documented, including hydrogen peroxide (H_2_O_2_), which breaks seed dormancy via phytohormonal regulation [64,65]. However, seed germination necessitates well-balanced ROS production and scavenging levels [66], as excessive accumulation of H_2_O_2_ could still be detrimental to a variety of cellular components, including nucleic acids, proteins, and membrane lipids [65]. Antioxidants, including catalase and SOD, transform ROS to H_2_O and O_2_ to provide better germination conditions. IAA production was also assessed in this study, with IAA application known to benefit GR, speed, and index [67].

As shown in Figure 3, the inoculation of isolates KW05 and KW07 on gibberellin-deficient Waito-C rice resulted in significantly improved growth. This finding underscores the growth-promoting mechanism of these isolates, likely attributed to GA secretion to the rhizosphere, as reported in previous studies [68,69]. Figure 4 illustrates GA production, with KW05 and KW07 showing higher production levels of GA_7_ and GA_4_, respectively. GAs regulate germination under unfavourable conditions [70] [71], promoting radicle emergence by stimulating hydrolytic enzymes to rupture the seed coat [72]. GAs also influence Na^+^ regulation and decrease the content of other ions [73]. GAs are pivotal in ensuring favourable GRs [74], as evidenced in studies on *Arabidopsis thaliana*, in which germination was significantly reduced upon application of paclobutrazol (a GA biosynthesis inhibitor), and on *Brassica parachinensis*, where the suppression of GA_4_ through exogenous coumarin application significantly reduced germination rate [75].

Isolates KW05 and KW07 were inoculated onto mallow and broccoli seeds with notable outcomes (Table 2). Inoculation significantly enhanced germination performance during salt stress compared with uninoculated seeds. Figure 6 shows that KW05 inoculation increased the total length of mallow and broccoli by 38.61% and 3.91%, respectively, during salt stress. Additionally, KW07 improved fresh weight and seedling length for both mallow and broccoli by 32.04%, 25.50%, 28.79%, and 19.34%, respectively. High salt and ROS levels under stress likely hindered germination and growth due to their toxicity, with similar findings reported previously [76]. However, inoculating KW05 and KW07 mitigated these adverse effects, likely due to their GA production, which supports germination under unfavourable conditions. These findings are consistent with a previous study [77] where the application of GA-producing isolates *Pseudomonas aeruginosa* and *Bacillus megaterium* yielded positive effects on seed germination in pigeon pea plants. Similarly, inoculation with GA-producing *Pseudomonas putida* ameliorated soybean seedling growth effects under saline conditions [78]. The observed improvements in germination and seedling growth were likely driven by the isolates’ production of siderophores, EPS, and antioxidants, as well as their phosphate-solubilizing activities. These findings align with various studies, where the aforementioned metabolites and activities were crucial in ensuring more favourable conditions for seed germination and seedling growth, particularly in high-salt environments [79,80,81]. There is limited research in Korea, where gibberellin-producing bacteria can enhance the growth and development of horticultural crops, especially in mallow and broccoli. Additionally, genomic and proteomic analysis is required to determine the suitability of these two gibberellin-producing isolates for enhancing the crop production on a larger scale in adverse conditions.

## 5. Conclusions

Previous studies have reported that there are only a few strains of PGPR that can produce gibberellin under salt stress. It is essential to identify the gibberellin producing PGPR from various sources and use them on plants experiencing different abiotic stresses. This study highlights the potential of rhizosphere soil isolates *Priestia aryabhattai* KW05 and *Pseudomonas frederiksbergensis* KW07 in broader applications to mitigate salt stress. The above-mentioned two isolates produced gibberellin, IAA, and an enhancement of antioxidant activities, which effectively promoted the germination of mallow and broccoli plant under salt-stress conditions. These findings indicate that gibberellin-producing bacteria may be a useful tool for alleviating salt stress in plants, which could be an ecofriendly approach for sustainable agriculture.

## Figures and Tables

**Table 1:** Screening of soil-derived bacterial isolates for several PGPR characteristics.

Bacterial Isolates	Geographical Coordinates of Soil Sample	SALKOWSKI TEST	Siderophore Production	Phosphate Solubilization	EPS Production	Identification
KW01	35.784548, 129.490484	++	+	+	*+*	*Streptomyces laurentii*
KW02	35.891247, 129.524323	+	+	+	*−*	*Priestia megaterium*
KW03	35.823135, 129.507986	+	−	++	*−*	*Priestia megaterium*
KW04	35.823135, 129.507986	+	+	++	*+*	*Priestia aryabhattai*
KW05	35.823135, 129.507986	+++	++	+++	*+*	*Priestia aryabhattai*
KW06	35.973071, 129.551715	+	+	+	*−*	*Bacillus velezensis*
KW07	35.951894, 129.543674	++	++	+++	*+*	*Pseudomonas frederiksbergensis*

Notes: Salkowski test, siderophore production, phosphate-solubilizing index, and EPS (exopolysaccharides) production: − (no), + (low), + + (moderate), + + + (high).

**Table 2:** Effects of KW05 and KW07 inoculation on the germination metrics of mallow and broccoli seedlings under no- and salt-stress conditions. Data represent means ± SDs from three replicates. Shared letters show statistical similarity, whereas different letters indicate significant differences followed by Duncan’s multiple range test at a significance level of *p* ≤ 0.05.

Crops	Treatment	PG (%)	GE (%)	GR (%/Day)	MGT (Day)	MDG	GPI
Mallow	NS	70 ^ab^ ± 5.8	70 ^a^ ± 5.8	5.89 ^a^ ± 1.09	1.41 ^d^ ± 0.21	1.4 ^ab^ ± 0.12	53.5 ^a^ ± 13.4
	KW05	80 ^ab^ ± 10	63.3 ^ab^ ± 6.7	5.05 ^ab^ ± 0.73	2.20 ^c^ ± 0.02	1.6 ^ab^ ± 0.2	36.2 ^ab^ ± 4.3
	KW07	83.3 ^a^ ± 3.3	60 ^ab^ ± 15.3	5.46 ^ab^ ± 0.78	2.31 ^c^ ± 0.44	1.67 ^a^ ± 0.07	40.2 ^ab^ ± 11.1
	SS	63.3 ^b^ ± 3.3	33.3 ^c^ ± 3.3	2.77 ^c^ ± 0.21	2.83 ^a^ ± 0.10	1.07 ^c^ ± 0.07	22.5 ^c^ ± 1.9
	SS + KW05	63.3 ^b^ ± 3.3	46.7 ^b^ ± 3.3	3.35 ^b^ ± 0.14	2.63 ^b^ ± 0.06	1.27 ^b^ ± 0.07	26.3 ^b^ ± 1.5
	SS + KW07	66.7 ^ab^ ± 3.3	53.3 ^b^ ± 3.3	3.64 ^b^ ± 0.19	2.62 ^ab^ ± 0.19	1.40 ^ab^ ± 0	25.6 ^b^ ± 1.6
Broccoli	NS	96.7 ^a^ ± 3.3	96.7 ^ab^ ± 3.3	9.50 ^ab^ ± 0.29	1.03 ^c^ ± 0.03	1.93 ^a^ ± 0.07	93.6 ^a^ ± 3.2
	KW05	100 ^a^ ± 0	100 ^ab^ ± 0	9.83 ^a^ ± 0.17	1.03 ^c^ ± 0.03	2.00 ^a^ ± 0	97.0 ^a^ ± 3.0
	KW07	96.7 ^a^ ± 3.3	96.7 ^ab^ ± 3.3	9.50 ^ab^ ± 0.50	1.04 ^c^ ± 0.04	1.93 ^a^ ± 0.07	93.7 ^a^ ± 6.3
	SS	96.7 ^a^ ± 3.3	90 ^b^ ± 0	7.33 ^c^ ± 0.14	1.65 ^a^ ± 0.11	1.93 ^a^ ± 0.07	58.9 ^c^ ± 1.9
	SS + KW05	96.7 ^a^ ± 3.3	96.7 ^ab^ ± 3.3	8.72 ^b^ ± 0.39	1.24 ^b^ ± 0.03	1.93 ^a^ ± 0.07	78.0 ^b^ ± 2.8
	SS + KW07	100 ^a^ ± 0	100 ^a^ ± 0	9.33 ^ab^ ± 0.33	1.13 ^bc^ ± 0.07	2.00 ^a^ ± 0	88.9 ^ab^ ± 5.6

Notes: Salt stress (SS), percent germination (PG), germination energy (GE), germination rate (GR), mean germination time (MGT), mean daily germination (MDG), and germination performance index (GPI).

## Data Availability

The data presented in this study are available in the article.

## Abbreviations

PG	Percent germination
MGT	Mean germination time
GR	Germination rate
GPI	Germination performance index
GE	Germination energy
MDG	Mean daily germination
